# Influence of Occupational Stress on the Body Mass Index of Hospital Workers: A Systematic Review

**DOI:** 10.3390/nu15183944

**Published:** 2023-09-12

**Authors:** Carlos Rodrigo Nascimento de Lira, Rita de Cássia Akutsu, Lorene Gonçalves Coelho, Karine Brito Beck da Silva, Jacqueline Costa Dias Pitangueira, Renata Puppin Zandonadi, Priscila Ribas de Farias Costa

**Affiliations:** 1 School of Nutrition, Federal University of Bahia, Avenida Araújo Pinho, n°32, Canela, Salvador 40110-150, Brazil; 2 Department of Nutrition, Campus Darcy Ribeiro, University of Brasilia, Asa Norte, Distrito Federal, Brasília 70910-900, Brazil; 3 Health Science Centre, Federal University of Recôncavo of Bahia, Avenida Carlos Amaral, n°1015, Cajueiro, Santo Antônio de Jesus 44430-622, Brazil

**Keywords:** occupational stress, hospital, body mass index

## Abstract

This systematic review aimed to identify the influence of occupational stress on the body mass index of hospital workers. After registering the protocol at PROSPERO (CRD42022331846), we started this systematic review following a search in seven databases, gray literature, as well as manual search and contact with specialists. The selection of studies was performed independently by two evaluators following the inclusion criteria: observational studies evaluating adult hospital workers, in which occupational stress was considered exposure and body composition as a result. The risk of bias in the included studies was assessed using the Joanna Briggs Institute Critical Appraisal checklist. We used the Grading of Recommendations Assessment, Development and Evaluation to grade the certainty of the evidence. Qualitative results were presented and synthesized through a qualitative approach, with simplified information in a narrative form. A total of 12 studies met the eligibility criteria and were included. This review comprised 10,885 workers (2312 men; 1582 women; and 6991 workers whose gender was not identified). Ten studies were carried out only with health workers, and two included workers from other sectors besides health workers. This review showed a relationship between occupational stress and changes in body mass index in hospital workers. However, most studies presented a moderate or high risk of bias and low quality of the evidence. These findings can be useful for clinical practice, administrators and leaders and provide insights for future research in the field of worker health in the hospital setting.

## 1. Introduction

Many health outcomes result from different exposure factors, and work processes, in many ways, contribute to shaping workers’ inadequate lifestyle habits and even illness. In this sense, precarious working conditions over the years have led to the scientific and legislative discussion on occupational stress [1,2,3].

Although the main problems related to the health of hospital workers, especially health professionals, are linked to infectious agents (e.g., hepatitis, influenza, tuberculosis, etc.), other factors also harm these workers’ health, such as risks arising from the workplace, long hours, standing posture, irregular meal times, and access to foods with low nutritional quality, among others. Thus, high psychological demand, physical exhaustion, stress and violence are documented in the scientific literature as striking aspects of this work environment and, in the medium to long term, contribute to the development of risk factors for non-communicable chronic diseases (NCD) [4,5,6].

Occupational stress in this study is addressed by the International Labor Organization [1] definition, which understands it as a harmful result of work demands that were not overcome by the individuals’ perceived resources and skills. The magnitude of this problem is worldwide, regardless of the country’s degree of development or the type of work carried out. However, even though occupational stress is a reality in all work activities, it occurs to a greater or lesser extent in certain groups of workers than in others [6,7].

The prevalence of occupational stress identified in many countries reinforces that it is a public health problem and a global concern [8,9,10,11,12,13]. Occupational stress and psychological issues contribute to changes in lifestyle, and food consumption is one of the issues addressed in studies associated with the emergence of NCD and changes in body composition [14,15,16,17,18]. For some authors, occupational stress, nutritional status and health conditions are such intertwined issues in the world of work that it only takes one of these to be out of adjustment to impact the others [5,19,20,21].

Previous reviews have assessed the relationship between occupational stress and the risk of type II diabetes mellitus [22]; occupational stress, change in stress and risk of weight gain and obesity [23]; obesity and stress in workers from different sectors of production [24]. A review evaluated the components, factors and results of occupational stress in nurses in studies published between 2009–2019 [25]. However, these reviews did not focus on hospital workers and, when they did, they did not include workers in sectors other than healthcare.

Given the lack of consensus on the relationship between occupational stress and body composition and the heterogeneity of the distribution of excess weight among different work occupations, where certain jobs present a higher risk of the worker becoming obese [7], understanding this dynamic is highly relevant both for the worker’s health and productivity and, consequently, for the growth of the organization. In this sense, this systematic review aimed to identify the influence of occupational stress on the body mass index of hospital workers.

## 2. Materials and Methods

### 2.1. Protocol and Registration

A systematic review protocol was prepared according to the Preferred Reporting Items for Systematic Review and Meta-Analysis Protocols—PRISMA-P [26] and submitted to the International Prospective Register of Systematic Review—PROSPERO platform [27], available under registration CRD42022331846. Therefore, this systematic review followed the recommendations of the Preferred Reporting Items for Systematic Reviews and Meta-Analyses—PRISMA [28] to answer the following question: “What is the influence of occupational stress on the body composition of hospital workers?

### 2.2. Eligibility Criteria and Search Strategy

Given the question posed for this review and based on the PECO acronym, the population included hospital workers; the exposure was occupational stress; the comparator, those workers without occupational stress; and the outcomes included were those referring to body composition.

The search was conducted in the Latin American and Caribbean Health Sciences Literature (LILACS), Cumulative Index to Nursing and Allied Health Literature (CINAHL), Medline/PubMed, Embase, Web of Science, Scopus and PsycINFO databases. As gray literature, Google Scholar was used in addition to manual search of the reference list of eligible studies and reviews identified in the search and contact with reference authors on the subject in question requesting studies that possibly were not retained in the searches.

The definition of descriptors and keywords was established from the acronym PECO, therefore considering exposure and outcome. The MeSH terms for Exposure (“Occupational Stress”) and Outcome (“Body Composition”, “Body Weight”, “Body Mass Index”, “Waist Circumference”, “Abdominal Circumference”, “Obesity, Abdominal”, “Waist- Hip Ratio”, “Weight Gain”, “Body Fat Distribution”, “Weight Loss”, “Body Weight Changes”, “Obesity”, “Sagittal Abdominal Diameter”) were combined with the Boolean operators AND and OR. The Emtree Thesaurus and Health Sciences Descriptors (DeCS) were respectively used for the Embase and LILACS databases. To increase search sensitivity, synonyms and similar terms were used. There were no restrictions regarding the year of publication, language, journal of publication, or authors’ affiliation.

Following the established procedure, the eligibility criteria adopted were I—Observational studies (cross-sectional, case-control and cohort), excluding reviews, instrument validation studies, intervention, communications, editorials, book chapters and studies with qualitative analysis; II—Only hospital workers: aged ≥ 18 years, regardless of the sector of work, employment relationship and work condition to which they were subjected, and both sexes; III—Occupational stress as exposure, as long as it is assessed by any validated instrument; IV—Studies where the outcome was body composition (fat mass, fat-free mass, body mass index—BMI, waist circumference, hip circumference, waist-to-hip ratio and percentage of total body fat), regardless of the method adopted for their classification and measurement.

### 2.3. Study Selection and Data Extraction

The search results were exported to EndNote^®^ Reference Manager (Clarivate Analytics, Philadelphia, PA, USA) in the online version, and the selection, after removing duplicates, was performed using the Rayyan^®^ online software (https://www.rayyan.ai/) (Rayyan, Qatar Computing Research Institute, Doha, Qatar) by two reviewers independently by reading titles and abstracts (Phase I). The articles that met the eligibility criteria or where there was still doubt as to their inclusion were read in full (Phase II). A third reviewer was contacted for a consensus meeting, if necessary. The selection process is detailed in a flowchart based on PRISMA [28].

Two independent reviewers extracted data from each original article in a spreadsheet prepared in Microsoft Office^®^ Excel version 2019. The extracted data included specific details about the publication (author; year of publication; country; sample size), participants (age; gender; marital status; years of work; working hours; work shift), the outcome of interest (values of association measures; mean; standard deviation; median; interquartile range; *p*-value; confidence interval), methods of study (study design; adopted analysis; methods to assess body composition), main limitations and conclusions. Missing data or additional information were requested by e-mail to the authors.

### 2.4. Analysis of Methodological Quality, Risk of Bias and Certainty of Evidence

Two reviewers independently assessed methodological quality using the Newcastle-Ottawa Scale (NOS). The NOS is divided into three blocks (selection, comparability and outcome) for cross-sectional and cohort studies and three blocks for case-control studies (selection, comparability and exposure). To classify the methodological quality, we used the recommendation of Sharmin et al. [29], which considers three or four stars as good methodological quality in the selection dimension, one or two in the comparability dimension, and two or three stars in the outcome dimension.

The risk of bias was assessed using the Joanna Briggs Institute Critical Appraisal checklist for cross-sectional and cohort studies. Included articles were classified as having “high risk of bias” when the study’s “yes” score was between 0% and 49%; “moderate risk of bias” when the study was scored “yes” between 50% and 69%; “low risk of bias” when the study “yes” score was ≥70% [30]. Judging was also performed by two reviewers independently, and a third reviewer resolved disagreements. The risk of bias result is presented descriptively, and RevMan 5.4.1 software (Review Manager 5.4, The Cochrane Collaboration, Oxford, UK) was used to create the figures.

The Grading of Recommendations Assessment, Development and Evaluation—GRADE was used to grade the certainty of the evidence [31]. A Summary of Results (SoF) table was created using the online software GRADEpro (https://www.gradepro.org/) (GRADE working group, McMaster University, Ontario, Canada) [32].

### 2.5. Data Synthesis Strategy

The strategy adopted for synthesizing qualitative data was a qualitative approach, with information presented in a narrative form to summarize and explain the findings. The results of the studies were not combined through meta-analysis due to the considerable heterogeneity between the studies [33].

### 2.6. Selection Process

With the search performed in the databases and gray literature, 5614 citations were retrieved. In addition, we contacted 12 authors considered experts on the subject; of these, only four responded. After their responses’ evaluation, we considered a study to integrate our review, totaling 5615 citations. Of these, 1361 were duplicates and were excluded, with only 4254 citations proceeding to phase I selection. After reading titles and abstracts (phase I), 4218 citations did not meet the eligibility criteria and were excluded. In phase I, we identified review studies that addressed the same theme (*n* = 9) and consulted their references. The title and abstract of one of the studies met the eligibility criteria and were included for complete reading in phase II (*n* = 37).

**Figure 1:**
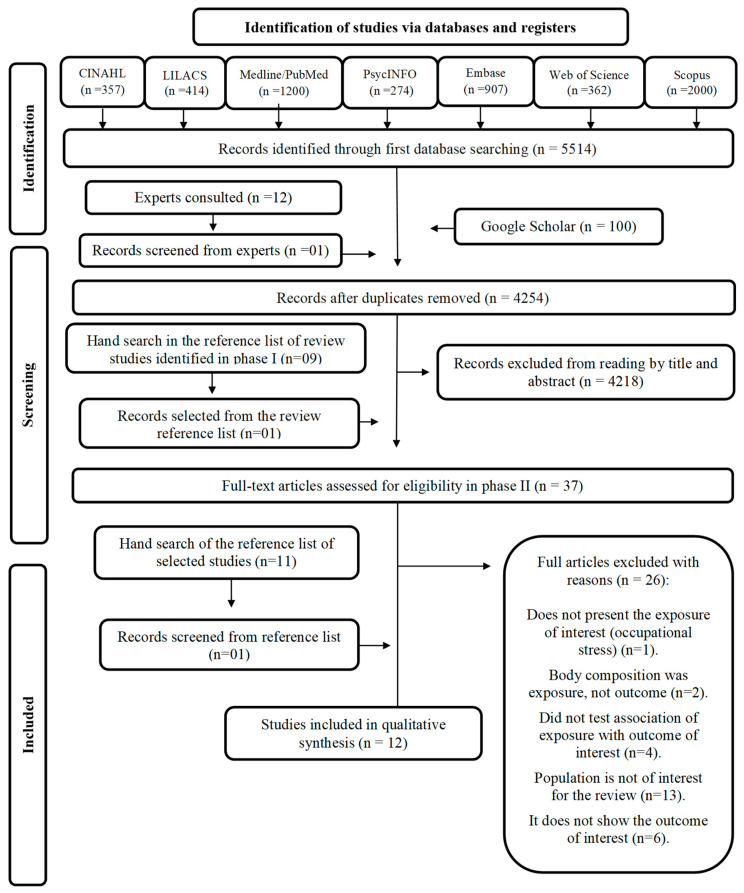
PRISMA flowchart describing the study selection process.

The 37 complete texts were read in full to verify their eligibility. As a result, 26 studies were excluded and 12 were considered in this review [34,35]. The PRISMA flowchart of the screening process is illustrated in Figure 1.

### 2.7. Characteristics of the Studies

The year of publication of the studies ranged from 2005 [36] to 2022 [37]. The 12 studies included were carried out in nine countries: two in Brazil, two in Taiwan, two in China and one in each of the following countries: Iran, Japan, India, Finland, Serbia and the United States of America. Of the studies, only one was longitudinal, and the others were cross-sectional. Only one was a postgraduate work (doctoral thesis), and the other articles were peer-reviewed. One article was published in Persian [34], one in Brazilian Portuguese [38] and the others in English (Table 1).

The declared sample in the included studies ranged from 33 to 6737 workers. Thus, this review is composed of 10,885 hospital workers, and among the studies that reported the sex of the sample, 2312 were men, 1582 were women and 6991 workers were not identified as to gender; two studies included only women in the sample (Table 1). Among the studies that presented the marital status of the participants, most of the participants were married (≥50%), and only in one study, 60.40% were single [38].

Regarding the jobs investigated in the studies, 10 were carried out only with health workers (with nursing professionals being the majority), and two studies also included workers from other hospital sectors (Table 1). In the study by Bardhan et al. [39], 21% of the sample worked in hospitals for more than 10 years. In Chou et al. [41] and Zhang et al. [35], 49.36% and 14.83% of participants had worked for more than 20 years, respectively. In the studies by Belkić and Nedic [40] and Fang et al. [42], the mean years were 2.8 (SD = 0.8) and 8.17 (SD = 6.94), respectively.

### 2.8. Assessment of Occupational Stress and Body Composition

The instruments used to assess occupational stress were the Job Content Questionnaire (JCQ) developed by Karasek [46] (adopted by three studies) [36,37,41]; the Effort-Reward Imbalance (ERI), created by Siegrist [47] and used in three other studies [35,39,42]; and the Job Stress Questionnaire (JSQ), which was the instrument adopted in three other studies [43,44,45], two of which used the short version, i.e., the Brief Job Stress Questionnaire (BJSQ). Of the three remaining studies, each one used a different instrument: Occupational Stress Index (OSI) [40], the Osipow Job Stress Questionnaire [34] and the Bianchi Stress Scale [38] (Table 1).

Regarding the outcome of interest for this review, only Coelho et al. [37] assessed more than one body composition indicator or proxies (waist circumference, BMI and body fat percentage); the other studies used only the BMI. Of these, three studies used participants’ self-reported weight and height to calculate BMI [36,39,40], one did not report how BMI was obtained [45] and in another, this information was unclear [35].

## 3. Results

### 3.1. Individual Study Results

The association evaluated in this review was tested and presented in different ways across studies. The only longitudinal study was also the only one showing body composition in addition to the use of proxies [37]. Findings identified that, in adjusted models, increased rates of high-level occupational stress were not significantly associated with any change in outcomes over time for abdominal obesity (OR = 0.74; 95%CI: 0.36–1.86), obesity by body fat percentage (OR = 1.74; 95%CI: 0.81–3.75) and obesity based on BMI (OR = 1.89; 95%CI: 0.76–4.72).

In the thesis by Barbosa [38], who studied 61 nurses at a university hospital in Rio de Janeiro/Brazil, the author found that only one (0.99%) of the evaluated subjects had a high level of stress and 39 of them (64.36%) a middle level. In the correlation analysis, a positive but not statistically significant correlation was found between BMI and the final score of the scale used to assess stress (r = 0.402; *p* = 0.084).

Abolfazli et al. [34] analyzed 142 nurses from the Ghaem Hospital in Iran and found that 82% of those studied had moderate to severe occupational stress and 34% only severe stress. A positive (but not statistically significant) correlation was found between overweight and occupational stress in the evaluated nurses (r = 0.023; *p* > 0.05).

In the study by Bardhan et al. [39] with 42 American nurses, occupational stress was measured from the effort-reward ratio and ability to deal with high work demands. Thus, 93% of these reported high stress levels (ERR > 1), and 83% were highly capable of dealing with high demand at work, where the cutoff point was 50. Through logistic regression analysis, an association was identified between obesity (OR = 2.653; 95%CI: 0.026–34.15) and overweight (OR = 1.731; 95%CI: 0.208–14.39) with high ERR (>1). In addition, obesity (OR = 2.006; 95%CI: 0.226–17.77) and overweight (OR = 1.684; 95%CI = 0.223–12.727) were also associated with high ability to deal with high work demands (>50).

A study with physicians in Serbia, Belkić and Nedic [40] did not present the percentage of occupational stress in the sample but only the average value of the variation of the total scores of the instrument used, the OSI, which was 77.3 (SD = 11, 8). A regression analysis model containing the total OSI score found significance (OR = 1.09; 95%CI: 1.02–1.16) to explain a BMI ≥ 28 kg/m^2^, and another model containing only two aspects of the OSI (avoid threats + conflict) also produced the best model to explain obesity, considered as BMI ≥ 30 kg/m^2^ (OR = 1.29; 95%CI: 1.07–1.55).

Chou et al. [41] evaluated 1329 Taiwanese hospital workers, and 19.71% of them (*n* = 262) reported high stress at work. Through a multivariate analysis, BMI was not correlated with work demand (regression coefficients = 0.007; *p* > 0.05), work control (regression coefficients = −0.001; *p* > 0.05) and work stress (regression coefficients = 0.000; *p* > 0.05). Also in Taiwan, Fang et al. [42] evaluated 237 participants, of which 20.5% (*n* = 49) had an effort/reward ratio > 1, reflecting high stress at work. There was an association between overweight/obesity and stress at work (*p* < 0.01), and overweight/obesity was highly associated with social support (t = 3.924; *p* < 0.01). In the multivariate logistic regression analysis, the authors found that nurses with high stress at work were 5.76 times (Exp(B) = 5.764; *p* < 0.01) more likely to be overweight/obese and nurses with a reduction of 1 point in social support were 0.035 times more likely to be overweight/obese (Exp(B) = 0.967; *p* = 0.010).

Fernandes and Shinde [43], evaluating 262 health workers in a hospital in India, found 69.85% of them (*n* = 183) with work stress. Using Spearman’s correlation test, the overall comparison of the relationship between BMI and stress at work showed no correlation between grade I obesity (r = 0.227; *p* = 0.265) and grade II obesity (data did not converge).

He et al. [44], evaluating 316 doctors and nurses in a hospital in China, found a prevalence of 23.7% of high stress and 18% of overload. Statistically significant positive correlations were also found between BMI and stress at work (r = 0.121, *p* < 0.05). Also in China, Zhang et al. [35] included in their study 1396 workers in the medical radiation sector (834 physicians, 208 nurses, 320 radiologists and 34 belonging to other positions) and identified that 741 of them (53.08%) had effort-reward imbalance greater than 1. The chi-square test demonstrated a statistically significant correlation between work strain and obesity among the evaluated team (χ^2^ = 20.647; *p* = 0.001).

Kouvonen et al. [36] evaluated the relationship between job stress and BMI among 45,810 Finnish workers. The authors did not report the prevalence of occupational stress among participants and identified that greater work demands (regression coefficient = 0.03; *p* < 0.05) and greater strain (regression coefficient = 0.02; *p* < 0.05) were significantly associated with higher BMI among nurses. However, high work control (regression coefficient = −0.01; *p* > 0.05) and greater effort-reward imbalance (regression coefficient = 0.01; *p* > 0.05) were not associated.

In Japan, Tsuboi et al. [45] investigated 33 nurses who worked at Fujita Health University Hospital; of these, 18 had high stress at work and 15 had low stress at work. The Student’s *t*-test identified no statistically significant association between BMI and occupational stress among the evaluated workers (t = −0.03; *p* > 0.05).

### 3.2. Assessment of Risk of Bias, Methodological Quality and Assessment of the Certainty of the Evidence

**Figure 2:**
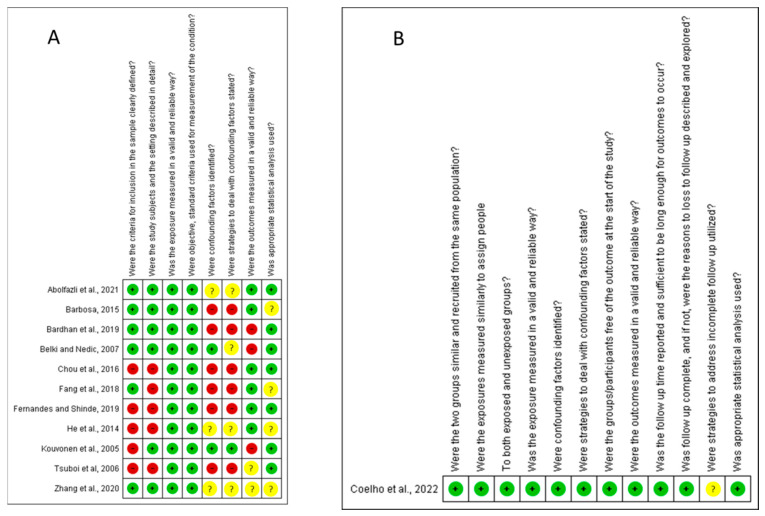
Authors’ judgments for each included study, as assessed by the JBI Critical Appraisal Checklist for Analytical Cross-Sectional Studies (**A**) and JBI Critical Appraisal Checklist for Cohort Studies (**B**). Subtitle: Abolfazli et al., 2021 [34]; Barbosa, 2015 [38]; Bardhan et al., 2019 [39]; Belkić and Nedic, 2007 [40]; Chou et al., 2016 [41]; Coelho et al., 2022 [37]; Fang et al., 2018 [42]; Fernandes and Shinde, 2019 [43]; He et al., 2014 [44]; Kouvonen et al., 2005 [36]; Tsuboi et al., 2006 [45]; Zhang et al., 2020 [35]. Green = low risk of life; red = high risk of life; yellow = moderately life-threatening.

Of the 12 included studies, 33.33% had a low risk of bias, 50% had a moderate risk and 16.67% had a high risk of bias. The items that most commonly increased the risk of bias between studies were the following: sample inclusion criteria were not clearly defined; the study subjects and setting were not described in detail; no confounding factors were identified; and strategies for dealing with confounding factors were not stated. More information about the risk of bias is found in Figure 2.

Concerning the methodological quality of the 12 studies included in this systematic review, all had good methodological quality, reaching six [38] to nine stars [40], according to the score suggested by Sharmin et al. [29] (Table 2). Internal validity was little affected, as only one study [45] did not report how BMI was assessed, and all studies assessed occupational stress using highly validated instruments. External validity, on the other hand, was limited due to the sampling process [39,41,43,44,45], as there was no declaration of sample calculation or lack of description of the response rate or the characteristics of subjects who responded and those who did not respond [34,38,43] (Table 2).

In addition, external validity also demonstrates limitations, as two studies [42,45] included only women. In addition, studies were collected in only three continents: America (Brazil and USA), Europe (Finland and Serbia) and Asia (China, Iran, India, Japan and Taiwan).

The Assessment, Development and Evaluation Classification of Recommendations (GRADE) was performed for the outcomes BMI, waist circumference and body fat (Table 3). For BMI, confidence was considered very low, and for the other outcomes, low, according to the GRADE criteria. The risk of bias assessment was the domain that presented the most problems, followed by inconsistency, due to the high heterogeneity.

## 4. Discussion

In this systematic review, we identified an association between occupational stress and excess weight in hospital workers based on BMI. However, caution is needed when interpreting this finding. The first point of reflection is that body composition is understood as the total body mass of an individual, and its evaluation is established by dividing the components that make up the human body into proportions, that is, fat mass (visceral fat and subcutaneous fat) and fat-free mass (muscles, bones, organs, ligaments, tendons and water) [48]. Therefore, the evidence provided in this systematic review points out that the fact that only one of the included studies uses body composition as it is understood and the others use only proxies for body composition based on anthropometric methods is a limitation of the studies when evaluating the body composition of these workers.

Also, in addition to the studies evaluating the association between occupational stress and anthropometric outcomes, the only indicator used was BMI. Despite its low cost and easy application and interpretation as positive points, it is known that this index only considers the ratio between the individual’s weight and height squared. Therefore, its major limiting factor is that it does not assess the composition and distribution of body compartments [48]. Therefore, it is not possible to distinguish between weight derived from fat or fat-free mass. Thus, we reinforce that, even though anthropometry is recognized as an efficient method for assessing health and disease and is widely used in epidemiological studies, it is necessary to advance research on body composition.

An alternative for researchers and professionals who assess workers’ nutritional status is to use more than one anthropometric indicator to provide robustness in diagnosing anthropometric status. With the unavailability of costly methods (e.g., bioimpedance, resonance, etc.) to assess body composition, anthropometric measurements have many advantages, such as being easy to apply and interpret, being non-invasive, requiring less preparation by individuals and having less cost for the service [49]. Therefore, they can be frequently repeated and it becomes easier to manage the logistics in epidemiological studies or the medical services of companies. On the other hand, researchers should be aware of the limitations it presents, such as low sensitivity to detect changes in the short term, lack of evaluator experience, low accuracy in obese individuals (in the use of skinfolds, for example) and failure to identify deficiencies in specific nutritional requirements [49].

The exposure of interest for this review, occupational stress, can be assessed in a perceived way using questionnaires [50] or by blood cortisol levels [51]. In this sense, epidemiological studies tend to use instruments due to their low cost, easy applicability and greater participants’ adherence. In the articles included here, the use of validated instruments was unanimous and, when necessary, adapted to the culture of the country where the data collection occurred. These instruments can inform the presence or absence of occupational stress and also categorize the stress level. In this review, eleven studies reported workers’ stress degree; only Kouvonen et al. [36] did not report the degree of stress in 6737 Finnish nurses.

Occupational stress is a reality in all environments and jobs [9,52], and in hospitals with a heterogeneous workforce, it is no different. Among the jobs evaluated by the 12 studies that met the eligibility criteria, 10 were carried out only with health workers, thus denoting that these are still the main focus of investigations in the field of worker health in the hospital environment, and even among the health workers, there are still those who are little included, such as nutritionists, speech therapists and physiotherapists, among others.

The importance of health professionals in the hospital sector is unquestionable, especially the medical and nursing staff. However, workers in other positions are not exempt from factors that contribute to occupational stress and poor health outcomes. Only Chou et al. [41] and Coelho et al. [37] included workers from other hospital sectors in their sample, such as workers in the administration sector. This finding suggests that studies need to be expanded when addressing hospital workers, mainly because each sector within the organization has its labor dilemmas [53,54].

The presence of stress is not in itself a guarantee of the occurrence of disturbances or diseases, but it is the experience of exposure to stressors for a long period that will trigger in the individual the phase of exhaustion associated with adverse health effects [55]. In this sense, it is understood that the service developed in a hospital is uninterrupted and workers are immersed in a routine of long working hours, and rotating shifts that, added to the precariousness of work, end up keeping these workers at high stress levels, which can lead to excess weight.

It is estimated that occupational stress also affects other factors that influence the body mass index, such as eating habits and physical activity practice. It is noted that with the dynamics of stress, often the high consumption of food, the intake of high-calorie meals and having irregular meals more frequently are not perceived by the worker, and the non-regularity in the practice of physical activity or the sedentary lifestyle triggers high rates of overweight and obesity.

Although some studies included in the systematic review have suggested a positive association between occupational stress and excess weight, care is needed in its interpretation since, in addition to the factors already discussed above, 50% of the studies showed a moderate risk of bias and 16.67% high risk of bias; the shallow quality of the evidence found and the high heterogeneity limit these findings.

Among the issues that led to these biases, the outcome evaluated in this review was obtained in a way that requires caution, as there was no information on how the variables for calculating the BMI were obtained [45], there was unclear information about BMI obtained [35] or self-reported weight and height were used to calculate the outcome [36,39,40]. Although self-reporting is accepted in scientific research [56,57], caution is needed when interpreting the results, as self-deception and memory problems are related to this practice [58]. Del Duca et al. [59], when conducting a study in Brazil to assess the validity of self-reported weight and height measurements, also reinforce that the use of such information should be made with caution in population studies, especially those that intend to use these values as continuous variables and /or to test associations.

Still, of the 11 cross-sectional studies, three [36,41,45] did not declare the sample inclusion criteria. In one study [43], despite its reporting that there was an inclusion criterion, these criteria were not listed in the study methodology. Five studies did not describe their subjects and/or setting [41,42,43,44,45].

Another point that reverberated in assessing the risk of bias was the statistical analysis presented by the articles. Fang et al. [42], He et al. [44] and Zhang et al. [35] did not report whether a data normality test was performed; Fang et al. [42], Zhang et al. [35] and Barbosa [38] did not report how the covariates were selected for inclusion in the regression models; moreover, only Belkić and Nedic [40] and Kouvonen et al. [36] identified confounding factors, but only Kouvonen et al. [36] stated strategies to deal with these factors. Statistical analysis and description of data are an essential step in the dissemination of science; however, many manuscripts in the medical and public health area present a series of statistical errors and/or in reporting them, which were also observed in studies included here: showing results with *p*-value only and without confidence intervals; reporting “*p* = ns” or “*p* < 0.05” or other arbitrary bounds instead of reporting exact *p*-values; not discussing sources of potential bias and confounding factors, among many others [60,61]. Presenting only the *p*-value without any context or other evidence is too limiting, so authors need to contextualize the *p*-value found with parameters such as quality of study design, internal validity, confidence intervals, numerical summaries and data graphics, as well as reinforce the difference between statistical significance and clinical relevance [62,63].

The low and very low quality of the evidence found to support the evaluated results is also another issue that causes the findings of this review to be interpreted with caution. In assessing the quality of evidence and strength of recommendations through the GRADE system, observational studies start out as low-quality evidence [31], which is one of the reasons for the low rating identified here. In addition, the inconsistency was considered serious since high heterogeneity was presented as well as the risk of bias.

Given all the context presented, this review has some limitations, such as the fragility of the methodological approach of the studies, especially concerning the methods of assessing body composition. The design of the included studies is another important limitation, as 11 of the 12 selected studies have a cross-sectional design, and by nature, it is not possible to establish a temporal relationship between the studied variables, that is, it is impossible to establish any causal inference between occupational stress and body composition [64]. Therefore, longitudinal studies and/or clinical trials with long-term follow-up periods would improve the findings’ robustness and provide better-quality evidence [65].

Despite the imposed limitations, we consider that the positive points can qualify the findings presented here. We can highlight as positive points of this review: (1) the review carried out a systematic search in seven databases plus the gray literature; (2) the review searched in sources beyond the recognized ones, such as consultation with specialists, analysis of the reference list of included studies, search in previous reviews on occupational stress; (3) there was no restriction on geography, language or the year of publication of the studies; (4) and finally, another strong point of this review was the use of a robust and rigorous methodological approach at all stages of the review, carried out by independent authors, reducing the chance of study selection bias and ensuring rigor.

## 5. Conclusions

A relationship between occupational stress and changes in body mass index in hospital workers was found in this systematic review. However, we emphasize that caution is needed in its interpretation due to the risk of bias presented and the low quality of the evidence included.

Our findings may be helpful in clinical practice, since health professionals responsible for occupational medicine in organizations must be aware that workers who experience occupational stress may be at risk for changes in body mass index. Therefore, considering these stressors in assessing body composition during follow-up can help prevent chronic diseases linked to excess weight.

Also, as hospital work is surrounded by stressors inherent to the environment itself (for example, the relationship with patients and companions, bed turnover, interpersonal relationships between specializations, etc.), our results can be helpful for administrators and leaders to minimize those stressors based on the leadership/management model that is adopted and thus contribute to the good health of these workers.

In terms of research, because most studies focus exclusively on health workers, there is a need for more research on workers in sectors such as hygiene, security, food service, laundry, administration, archiving, stretcher bearers, etc. Thus, it is possible to identify how the demands of work in hospitals impact the body composition of these groups, as we know that in certain jobs, work can favor a sedentary behavior that, added to the demands, favors excess weight.

Therefore, we recommend that future studies, especially longitudinal and prospective ones, assess long-term changes concerning the occurrence of stress and the development of excess weight to increase the quality of the evidence. Furthermore, cross-sectional studies must use methods to assess body composition with a set of anthropometric indicators when using proxies so that it is possible to measure this variable. 

## Figures and Tables

**Table 1:** Summary of studies included in the systematic review, listed by publication, sample, exposure and outcome characteristics.

Author, Year	Country	Sample (Sex)	Age	Type of Worker	Diagnosis of Occupational Stress	Body Composition	Occupational Stress	Main Result
Abolfazli et al., 2021 [34]	Iran	142 (men/women)	20 to <50 years	Nurses Paramedics	Osipow Job Stress Questionnaire	BMI	Low: 1% Low to medium: 24% Moderate to severe: 82% Severe: 34%	Pearson’s correlation showed a positive relationship between overweight/obesity and stress at work (r = 0.023); however, a non-significant relationship (*p* > 0.05).
Barbosa, 2015 [38]	Brazil	61 (11 men; 50 women)	20 to 59 years	Nurses	Bianchi stress scale	BMI	Low: 34.65% Average: 64.36 High: 0.99%	The BMI variable was not associated with stress (r = 0.402; *p* = 0.084).
Bardhan et al., 2019 [39]	USA	42 (13 men; 29 women)	Average 33.04 years	Nurses	ERI	BMI	ERR ≤ 1: 7% ERR > 1: 93%	Obesity: (OR = 2.653, CI = 0.026–34.15); and overweight (OR = 1.731, 95%CI = 0.208–14.39) were associated with an ERR > 1.
Belkić and Nedic, 2007 [40]	Serbia	112 (men/women)	Average 48.9 years	Doctors	Occupational Stress Index	BMI	Mean = 77.3 (±11.8)	The total OSI explains the greatest variation for BMI ≥ 28 kg/m^2^ (OR = 1.09; 95%CI = 1.02; −1.16), whereas the sum of two OSI aspects [avoiding threats + conflict] produced the best model to explain obesity (OR = 1.29; 95%CI = 1.07; –1.55).
Chou et al., 2016 [41]	Taiwan	1329 (1101 men; 228 women)	≤30 to >50 years	Doctors Nurses Administrators	JCQ Chinese version	BMI	Low tension: 33.10% Passive work: 30.10% Active work: 17.09% High voltage: 19.71%	BMI did not correlate with work demand (regression coefficient = 0.007), work control (regression coefficient = −0.001) and work stress (regression coefficient = 0.000) *p* > 0.05.
Coelho et al., 2022 [37]	Brazil	218 (54 men; 164 women)	Average 32.6 years	Health professionals and administration	JCQ	BMI WC BF	Before 14.2% After 29.4%	Considering changes in the level of occupational stress during the observed period, increased rates of high-level occupational stress were not significantly associated with any change in outcomes over time (BMI *p* = 0.944; WC *p*= 0.971; BF *p* = 0.186).
Fang et al., 2018 [42]	Taiwan	237 (women)	Average 33.46 years	Nurses	ERI	BMI	ERR > 1: 20.5% ERR ≤: 78.7%	Overweight/obesity was associated with stress at work (*p* ≤ 0.01). The study showed that high stress at work (ERR > 1) was an important predictor of overweight/obesity. Nurses with high stress at work were 5.76 times (β = 5.764; *p* < 0.01) more likely to be overweight/obese.
Fernandes and Shinde, 2019 [43]	India	262 (59 men; 203 women)	Average 26.2 years	Dentists Doctors Nurses Pharmacists Physiotherapists	BJSQ	BMI	With stress: 69.85% No stress: 30.15%	The value of the correlation between BMI and stress at work in the normal (r = 0.006; *p* = 0.939), overweight (r = −0.022; *p* = 0.852) and obesity (r = 0.227; *p* = 0.265) category of BMI was weakly correlated with job stress.
He et al., 2014 [44]	China	316 (278 men; 38 women)	20 to ≥40 years	Doctors Nurses	JSQ	BMI	Relaxed: 12.3% Normal: 45.9% Too much stress: 23.7% Overload: 18%	Statistically significant positive correlations were found between BMI and stress at work (r = 0.121; *p* ≤ 0.05).
Kouvonen et al., 2005 [36]	Finland	6737 (men/women)	-	Nurses	JCQ	BMI	-	Greater work demands (standardized regression coefficient = 0.03; *p* ≤ 0.05) and greater strain (standardized regression coefficient = 0.02; *p* ≤ 0.05) were significantly associated with higher BMI among nurses.
Tsuboi et al., 2006 [45]	Japan	33 (women)	-	Nurses	BJSQ	BMI	Low stress: 45.5% High stress: 54.5%	There was no significant difference in BMI (unpaired *t*-test = −0.03; *p* ≥ 0.05) between workers with high (20.6 ± 1.86) or low stress (20.6 ± 1.68).
Zhang et al., 2020 [35]	China	1396 (796 men; 600 women)	<30 to ≥50 years	Radiation industry workers	ERI	BMI	ERI ≤ 1: 46.92% ERI > 1: 53.08%	There was a statistically significant difference in work stress in association with obesity (χ2 = 20.647; *p* ≤ 0.001).

Abbreviations: ERI = Effort-Reward Imbalance; OSI = Occupational Stress Index; JCQ = Job Content Questionnaire; JSQ = Job Stress Questionnaire; BJSQ = Brief Job Stress Questionnaire; ERR = Relação Esforço/Recompensa; BMI = Body Mass Index; WC = Waist Circumference; BF = Body fat; OR = Odd Ratio. Considering working hours, in the study by Bardhan et al. [39], 50% of the sample worked ≤40 h/week. In the study by Fang et al. [42], the working hours were <44 h/week for 55% of the workers and 38% had working hours of ≥44 and <48 h/week. The work shift in the study by Bardhan et al. [39] was daytime for 38% of workers and rotating for 62% of them. In the study by Chou et al. [41], 48% worked during the day, 3.46% at night and 48.54% worked shifts. In the study by Fang et al. [42], 66.5% of workers worked in rotating shifts.

**Table 2:** Methodological quality of studies included by the Newcastle-Ottawa Scale.

Cross-Sectional Study	Selection	Comparability	Outcome	Total
Abolfazli et al., 2021 [34]	* * * *	*	* * *	08
Barbosa, 2015 [38]	* * * *	*	*	06
Bardhan et al., 2019 [39]	* * * *	*	* *	07
Belkić and Nedic, 2007 [40]	* * * * *	*	* * *	09
Chou et al., 2016 [41]	* * * *	*	* * *	08
Fang et al., 2018 [42]	* * * * *	*	* *	08
Fernandes and Shinde, 2019 [43]	* * *	*	* * *	07
He et al., 2014 [44]	* * * *	*	* *	07
Kouvonen et al., 2005 [36]	* * * * *	*	* *	08
Tsuboi et al., 2006 [45]	* * * * *	*	*	07
Zhang et al., 2020 [35]	* * * * *	*	* *	08
Cohort Study	Selection	Comparability	Outcome	Total
Coelho et al., 2022 [37]	* * * *	*	* * *	08

* Refers to the number of points obtained in each component (Selection, Comparability, Result) of the Newcastle-Ottawa Scale.

**Table 3:** Summary of the Assessment, Development and Evaluation Classification of Recommendations (GRADE).

Certainty Assessment							Certainty
№ of Studies	Study Design	Risk of Bias	Inconsistency	Indirectness	Imprecision	Other Considerations	
Body Mass Index							
12	Observational studies	Serious ^a^	Serious ^b^	Not serious	Not serious	None	⨁◯◯◯ Very low
Waist Circumference							
01	Observational studies	Not serious	Not serious	Not serious	Not serious	None	⨁⨁◯◯ Low
Body Fat							
01	Observational studies	Not serious	Not serious	Not serious	Not serious	None	⨁⨁◯◯ Low

Explanations: CI: confidence interval. ^a^ Among the studies evaluated, only three had a low risk of bias and the others ranged from moderate to high risk of bias. ^b^ High heterogeneity.

## Data Availability

Not applicable.
